# Reclassification of two germline *DICER1* splicing variants leads to DICER1 syndrome diagnosis

**DOI:** 10.1007/s10689-023-00336-1

**Published:** 2023-05-30

**Authors:** Maria Apellaniz-Ruiz, Nelly Sabbaghian, Anne-Laure Chong, Leanne de Kock, Semra Cetinkaya, Elvan Bayramoğlu, Winand N. M. Dinjens, W. Glenn McCluggage, Anja Wagner, Aslihan Arasli Yilmaz, William D. Foulkes

**Affiliations:** 1 Genomics Medicine Unit, Navarrabiomed, Hospital Universitario de Navarra (HUN), Universidad Pública de Navarra (UPNA), IdiSNA, Calle Irunlarrea 3, 31008 Pamplona, Navarra Spain; 2 Lady Davis Institute, Segal Cancer Centre, Jewish General Hospital, McGill University, Montréal, QC Canada; 3 Children’s Hospital of Eastern Ontario Research Institute, Ottawa, ON Canada; 4 Department of Pediatric Endocrinology, Health Science University, Dr Sami Ulus Obstetrics and Gynecology, Children’s Health and Disease Training and Research Hospital, Ankara, Turkey; 5 Department of Pathology, Erasmus MC Cancer Institute, University Medical Center Rotterdam, Rotterdam, The Netherlands; 6 Department of Pathology, Belfast Health and Social Care Trust, Belfast, UK; 7 Department of Clinical Genetics, Erasmus MC Cancer Institute, University Medical Center Rotterdam, Rotterdam, The Netherlands; 8 Program in Cancer Genetics, Department of Oncology and Human Genetics, McGill University, Montréal, QC Canada; 9 Department of Medical Genetics, Research Institute of the McGill University Health Centre, Montréal, QC Canada

**Keywords:** *DICER1* syndrome, Splicing variant, Functional characterization, Sertoli-Leydig cell tumour, Multinodular Goitre, Variant classification

## Abstract

*DICER1* syndrome is an inherited condition associated with an increased risk of developing hamartomatous and neoplastic lesions in diverse organs, mainly at early ages. Germline pathogenic variants in *DICER1* cause this condition. Detecting a variant of uncertain significance in *DICER1* or finding uncommon phenotypes complicate the diagnosis and can negatively impact patient care. We present two unrelated patients suspected to have DICER1 syndrome. Both females (aged 13 and 15 years) presented with multinodular goiter (thyroid follicular nodular disease) and ovarian tumours. One was diagnosed with an ovarian Sertoli-Leydig cell tumour (SLCT) and the other, with an ovarian juvenile granulosa cell tumour, later reclassified as a retiform variant of SLCT. Genetic screening showed no germline pathogenic variants in *DICER1*. However, two potentially splicing variants were found, *DICER1* c.5365-4A>G and c.5527+3A>G. Also, typical somatic *DICER1* RNase IIIb hotspot mutations were detected in the thyroid and ovarian tissues. In silico splicing algorithms predicted altered splicing for both germline variants and skipping of exon 25 was confirmed by RNA assays for both variants. The reclassification of the ovarian tumour, leading to recognition of the association with DICER1 syndrome and the characterization of the germline intronic variants were all applied to recently described *DICER1* variant classification rules. This ultimately resulted in confirmation of DICER1 syndrome in the two teenage girls.

**Supplementary Information:**

The online version contains supplementary material available at 10.1007/s10689-023-00336-1.

## Introduction

DICER1 syndrome (OMIM #601200) is a rare tumour predisposition syndrome caused by pathogenic germline variants in *DICER1* [1]. A plethora of primarily early-onset neoplastic and hamartomatous lesions have been associated with DICER1 syndrome, including pleuropulmonary blastoma, thyroid follicular nodular disease (TFND), intestinal polyps and ovarian Sertoli-Leydig cell tumour (SLCT) [1, 2].

Typically, patients with DICER1 syndrome have a combination of a loss‐of‐function germline variant and a characteristic tumour-specific RNase IIIb hotspot missense mutation [1]. Whereas germline variants occur across the entire gene, the somatic mutations affect catalytically active metal‐ion binding residues (p.E1705, p.D1709, p.D1713, p.G1809, p.D1810, p.E1813) [1].

Identifying a germline pathogenic *DICER1* variant in an individual allows the clinicians to provide appropriate surveillance to prospectively screen for further *DICER1*-associated tumours and to offer genetic testing for all first-degree relatives [3, 4].

The problem arises when a variant of uncertain significance (VUS) is detected [5]. This is the case of intronic variants outside the canonical splice sites. However, applying in silico prediction tools to select candidate variants, coupled with experimental evaluation using in vitro minigene assays or analyzing patient’s RNA [5] allow a more accurate variant interpretation [6].

In this report, we present two cases of teenage females who appeared to have DICER1 syndrome based on the clinical presentation and the identification of somatic *DICER1* hotspot mutations. However, germline screening, variant segregation and phenotype attribution were not straightforward.

## Results

### Clinical phenotype of case 1

**Fig. 1:**
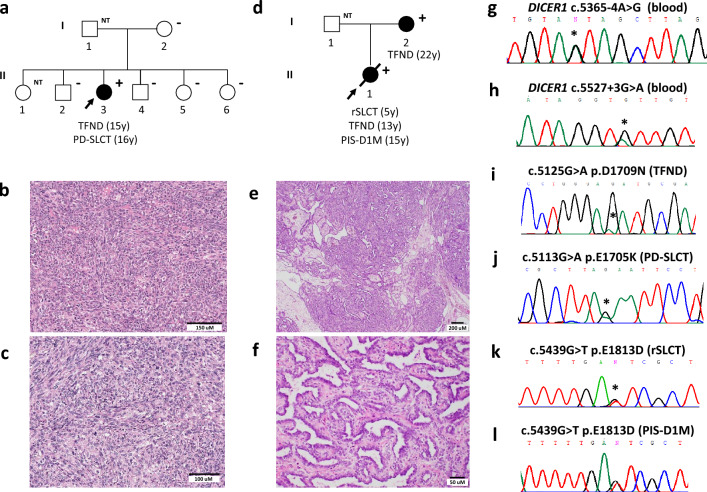
Family information, representative hematoxylin and eosin-stained photomicrographs of the ovarian tumours and *DICER1* Sanger sequencing results. Case 1: **a** Pedigree. **b, c** Images of the poorly differentiated SLCT showing **b** diffuse sheets of immature Sertoli cells with focal vague corded formation and **c** cells displaying high-grade nuclear atypia with bizarre forms. *DICER1* germline (**g**) and somatic (**i, j**) variants identified. Case 2: **d** Pedigree. **e, f** Images of the retiform SLCT. **e** Low-power image showing slit-like spaces, some of which are dilated. **f** High-power image showing slit-like spaces lined by cuboidal cells. *DICER1* germline (**h**) and somatic (**k, l**) variants identified. In the pedigrees: arrows indicate the proband; “NT” means not tested, “−” stands for wild type and “+” means heterozygous for the *DICER1* germline variant. Variants are marked with an asterisk. *DICER1* transcript NM_177438 was used for the nomenclature. *PIS-D1M* Primary Intracranial Sarcoma DICER1-mutant, *TFND* thyroid follicular nodular disease, *PD-SLCT* poorly differentiated Sertoli-Leydig cell tumour, *rSLCT* retiform Sertoli-Leydig cell tumour, *y* years

A 15-year-old female was admitted to a Paediatric Endocrinology department with menstrual irregularity, hirsutism and neck swelling. She was the third child of non-consanguineous parents and had an unremarkable medical history (Fig. 1a). There was no family history of disease, except the death of her maternal grandmother in her 30 s due to uterine cancer.

On physical examination, she had a thyroid goitre, loss of buccal fat pads, muscular appearance of the arms and legs, increased fatty tissue in the lower extremities and gluteal region, and hirsutism. Clinical workup revealed hyperandrogenism, insulin resistance, low leptin level (3.18 ng/ml), grade 1 hepatic steatosis and acquired partial lipodystrophy. Metformin treatment was started. Thyroid ultrasound showed multiple nodules with solid and cystic components, the largest being 2 cm. Bilateral near-total thyroidectomy was performed following a thyroid aspirate where follicular neoplasia was suspected. Pathology review of the thyroid-resected specimen was consistent with TFND and showed diffuse degenerative changes. Two months later, a new nodule developed and enlargement of the existing residual nodules was observed. Surgery was performed to completely remove the thyroid. Review of this specimen was also compatible with TFND.

One year later, the patient presented with menometrorrhagia. Despite metformin therapy, a significant increase in serum androgen and tumour marker levels were detected (AFP = 4.5 ng/mL, β-HCG < 2 mIU/mL, CA-125 = 26 IU/mL, CA-15–3 = 31.5 U/mL, CA-19–9 < 2.00 U/mL). An abdominal MRI showed a 7.5 cm right ovarian mass containing solid and cystic components. Right salpingo-oophorectomy, bilateral pelvic and paraaortic lymph node dissection and omentectomy was performed. Histopathological evaluation of the resected lesion showed a poorly differentiated SLCT (Fig. 1b). The tumour contained anaplastic foci with bizarre nuclei and atypical mitotic figures (Fig. 1c). No heterologous or retiform components were observed. Immunohistochemistry showed diffuse positivity with CD56, oestrogen receptor (ER) and progesterone receptor (PR) and focal staining with inhibin and calretinin. Four cycles of PEB protocol (bleomycin, etoposide and cisplatin) were administered. The patient is being followed and is currently in remission with no recurrence observed in pelvic MRI.

### Clinical phenotype of case 2

A 13-year-old female was referred to a department of Clinical Genetics due to suspicion of a genetic disorder, given her previous diagnoses of thyroid nodules and an ovarian tumour (Fig. 1d). Her parents were of Moroccan descent and non-consanguineous. The mother had a right total hemithyroidectomy and a left subtotal hemithyroidectomy at the age of 22 years. Histopathology analysis showed papillary thyroid hyperplasia. Family history of thyroid goitre on the maternal side was reported although clinical confirmation of disease or genetic testing was unavailable.

On physical examination, the adolescent had a normal head circumference, no syndromic features and no skin or mucous membrane aberrations. Medical history revealed she underwent emergency surgery for an ovarian torsion at the age of 5 years. The right ovarian mass was diagnosed as a juvenile granulosa cell tumour (JGCT), which was treated by surgical removal of the ovary and two cycles of chemotherapy (MAKEI-protocol). Immunohistochemical analysis showed the tumour was positive for inhibin but negative for calretinin. At the age of 13 years, she presented with multiple thyroid nodules. Fine needle aspirations only revealed benign lesions (Bethesda 2) and there was no definite indication for surgery. Based on clinical presentation, germline testing of *PTEN* and *DICER1* was performed. A VUS was identified in *DICER1*.

At the age of 15 years, the patient was admitted to hospital because of a large intraparenchymal cerebral hemorrhage. Seven months later, a second intracerebral bleed occurred. She developed hydrocephaly for which an external ventricular drain was placed, and later replaced by a ventriculoperitoneal drain. At that time, the patient was referred back to the Clinical Genetics department to evaluate a genetic cause of the intracranial hemorrhage. Aneurysm-related genes were screened using exome sequencing data obtained from germline DNA. However, no relevant genetic alteration was detected. Given the phenotypes identified in the patient and the presence of a VUS in *DICER1*, the possibility of DICER1 syndrome was revisited. Expert opinion on the pathogenicity of the *DICER1* VUS was sought and a histopathological review of the ovarian tumour by an expert gynecological pathologist (W.G.M) was undertaken. Histology showed a neoplasm with a low-power lobulated architecture and composed of bland epithelioid cells with a slit-like architecture. Small numbers of epithelioid cells with abundant eosinophilic cytoplasm were present. No heterologous elements were identified. There was focal positive staining with inhibin. Base on the morphology, the neoplasm was reclassified as a retiform variant of SLCT (Fig. 1e, f).

The patient clinically deteriorated, and an MRI showed a brain lesion compatible with a thrombosed aneurysm or a brain tumour. Emergency surgery was performed, and histopathological examination of the mass revealed a primary intracranial sarcoma, DICER1-mutant. The patient received radiotherapy and oral chemotherapy, but the sarcoma progressed, and she died at the age of 16 years.

### Molecular results

Given the association of TFND and SLCT with DICER1 syndrome [7, 8], germline and tumour DNA were subjected to *DICER1* screening. No pathogenic germline variant, complete exon deletion or gain in *DICER1* was detected in the patients (Supplementary Methods). Nevertheless, both individuals carried *DICER1* intronic variants in heterozygosity: case 1 harboured c.5365-4A>G in intron 24 (Fig. 1g) and case 2 carried c.5527+3A>G in intron 25 (Fig. 1h).

We then screened for somatic hotspot mutations. In case 1, c.5125G>A (p.D1709N) was identified in the TFND (Fig. 1i) and c.5113G>A (p.E1705K) in the SLCT (Fig. 1j). In case 2, the c.5439G>T (p.E1813D) hotspot mutation was detected in the retiform SLCT (Fig. 1k). No hotspot mutation was identified in the TFNDs from case 2 or from her mother’s. Molecular study of the brain sarcoma revealed somatic pathogenic mutations in *NF1* (c.4950del, p.Y1650* and c.6756+3A>C) and *TP53* (c.523C>G, p.R175G), as well as a somatic *DICER1* hotspot mutation (c.5439G>T p.E1813D) (Fig. 1l). DNA methylation revealed the tumour clustered in the group known as primary intracranial sarcoma, DICER1 mutant (score > 0.9).

Family segregation studies were conducted. The *DICER1* germline variant identified in case 1 was not detected in her mother nor in four of her siblings (Fig. 1a). Germline DNA from her father was not available to confirm a de novo origin. The variant identified in case 2 was detected in the patient’s mother, also diagnosed with TFND (Fig. 1d).

None of these variants appears in gnomAD v2.1.1 (revised in January 2023); however, c.5527+3A>G was catalogued in ClinVar (ID: 825804) and we submitted c.5365-4A>G (ID: 1713278).

Given intronic variants can alter canonical mRNA splicing, we ran five in silico splicing predictors (i.e. SpliceAI, Human Splicing Finder, NNSplice, NetGen2, dbscSNV). We obtained full concordance for c.5365-4A>G, predicting an acceptor splice site gain. However, not all tools predicted the loss of the donor splice site for c.5527+3A>G.

**Fig. 2:**
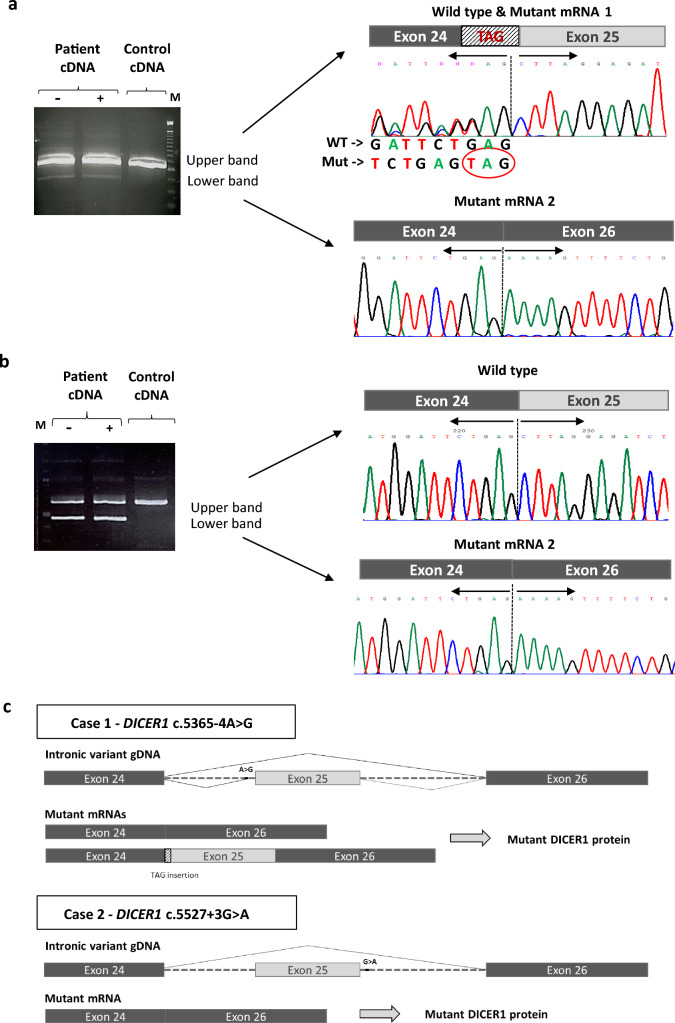
Effect of *DICER1* germline variants on mRNA splicing. Cropped images of agarose gels showing RT–PCR products in Case 1 (**a**) and in Case 2’s mother (**b**), demonstrating that c.5365-4A>G and c.5527+3G>A alter canonical splicing. Original images of the agarose gels are presented in Supplementary Figs. 3 and 4. On the right side, chromatograms showing the sequence of: **a** the wild type and two aberrant transcripts (mutant mRNA 1 & 2) detected in Case 1, and **b** the wild type and the aberrant transcript (mutant mRNA 2) found in Case 2’s mother. **c** Schematic representation of the gDNA and mRNAs in Case 1 and Case 2. *M* marker, *Mut* mutant transcript, *WT* wild type, − without cycloheximide, + with cycloheximide

We performed a splicing minigene assay with the pSPL3 vector to study the potential pathogenicity of *DICER1* c.5365-4A>G (Supplementary Methods). HEK293T cells were transfected with the constructs. Construct with c.5365-4A>G produced two aberrant transcripts, one skipping exon 25 and the other including 3 bases (TAG) before exon 25 (Supplementary Fig. 1). These results were later confirmed in the proband’s RNA (Fig. 2a). As expected, we detected wild type canonical transcripts, derived from the wild type allele, and two types of aberrant transcripts, derived from the altered allele (Fig. 2a). Cycloheximide treatment showed differences in mRNA transcription only on the transcript with the TAG insertion. This aberrant transcript seems to undergo nonsense mediated decay (NMD), at least to some extent (Supplementary Fig. 2). At protein level, the two aberrant transcripts would lead to a premature stop codon: p.L1789* when TAG is inserted, and p.L1789Kfs*42 with exon 25 deletion. These would result in truncated 1789 and 1830 amino acid long DICER1 proteins, respectively.

To study the effects of *DICER1* c.5527+3A>G on splicing (case 2), RNA was extracted from the mother’s fibroblasts (also heterozygous). RNA analysis revealed the presence of aberrant transcripts without exon 25, as well as wild type transcripts (Fig. 2b). Treating blood lymphocytes with cycloheximide ruled out NMD. In this case, the aberrant transcripts would lead to p.L1789Kfs*42 resulting in a DICER1 protein devoid of 92 amino acids.

Following the American College of Medical Genetics and Genomics and the Association for Molecular Pathology (ACMG-AMP) standards and guidelines for the classification of sequence variants and the gene-/disease-specific classification system developed by ClinGen DICER1 variant curation expert panel (VCEP) [6], we classified both *DICER1* intronic variants as likely pathogenic. c.5365-4A>G met PS3, PS4_Supporting, PM2_Supporting, PP3, PP4; and c.5527+3A>G met PS3, PS4_Moderate, PM2_Supporting, PP3, PP4. The criteria include well-established in vitro functional studies supportive of a damaging effect (PS3), increased prevalence in affected individuals (PS4), variant allele frequency < 0.000005 (PM2), in silico evidence supporting deleterious effect (PP3) and patient’s phenotype or family history highly specific for a disease, in this case, DICER1 syndrome (PP4) (additional details in Supplementary Information). Use of the new *DICER1* specific criteria has allowed us to be more precise and confident that the variant classification is correct.

## Discussion

Here, we report two young female patients with a suspicion of DICER1 syndrome. Given DICER1 syndrome confers increased risk of certain rare tumours [2, 3], the detection of one of these distinctive neoplasms or a combination thereof, should prompt genetic *DICER1* testing. Indeed, the presentation of thyroid nodules and SLCT is highly specific for DICER1 syndrome [1, 2, 7, 8]. However, the wide range of morphological features and overlap between ovarian sex cord-stromal tumours frequently pose diagnostic difficulties. This was the case of the ovarian tumour in proband 2, initially diagnosed as a JGCT. Moreover, finding a sarcomatous histology with rhabdomyoblastic differentiation in children, as in case 2, should also raise suspicion of *DICER1* [9], as well as observing papillary structures within a TFND, follicular adenoma or follicular carcinoma [10]. Therefore, expert histopathological evaluation in conjunction with molecular testing could be useful in establishing a correct diagnosis.

However, determining the clinical relevance of certain *DICER1* alterations can be challenging given the incomplete penetrance, variable expressivity and rarity of conditions [1]. In this study, given the lack of coding genetic variants in *DICER1* and taking into account the impact and high prevalence of splicing variants in disease, we looked for *DICER1* intronic variants. This allowed us to find one candidate in each patient. The allele frequency, family segregation and results from in silico predictors led us to perform RNA assays to assess the functional consequence of the VUSs. In both cases, the intronic *DICER1* variants altered the canonical splicing and produced mRNAs lacking exon 25. As the sequence of exons 24 and 25 encode the RNase IIIb catalytic domain, DICER1 proteins devoid of this domain may not be functional, if produced, or may be rapidly degraded (Fig. 2c). In this regard, it has been shown that the over-expression of a DICER1 Δexon25 construct in COS-1 cells can be translated, but less efficiently than the full-length DICER1 [11].

Applying the specific variant interpretation criteria developed by the ClinGen DICER1 and miRNA-Processing VCEP [6] to classify the pathogenicity of the germline *DICER1* intronic variants, we ultimately classified both variants as likely pathogenic. Consequently, surveillance strategies to follow these individuals and their relatives were applied to reduce DICER1-associated morbidity and mortality [3, 4].

This study has certain limitations such as the impossibility to obtain germline DNA from case 1’s father in order to establish if the *DICER1* variant detected in case 1 was inherited or de novo. Also, we did not identify somatic *DICER1* RNase IIIb hotspot mutations in the thyroid lesions from case 2 and her mother. This might be due to the thyroid tissue sampling. Another possibility is that a minority of nodules do not contain any detectable RNase IIIb mutations [12].

Obtaining a conclusive diagnosis is crucial for the patients and their family members. However, this is not always a straightforward path. This study serves as an example of one of the main challenges in clinical genetics, unveiling VUS, especially nowadays with the routine use of next generation sequencing techniques. Similarly to this work, a few reports have described *DICER1* intronic variants (either germline or somatic) in patients with phenotypes associated with DICER1 syndrome. However, in previous cases, a comprehensive analysis has not always been possible.

In conclusion, this report emphasizes the importance of expert histopathological review when facing atypical presentations, the identification of syndrome specific-associated diseases and/or hotspot mutations, the need to assess the pathogenicity of VUS, and the need for multidisciplinary team discussion to solve unusual cases.

### Supplementary Information

Below is the link to the electronic supplementary material.

Supplementary file1 (DOCX 2632 kb)

## Data Availability

Data generated and analysed during this study are included in this article and its supplementary information files. The germline *DICER1* variants studied here have been submitted to ClinVar (ClinVar accession numbers are SCV002589127 and SCV001372208.2; ClinVar variation IDs are 1713278 and 825804). Primer sequences and plasmids are available upon request.
