# Supplementary material for: Reclassification of two germline DICER1 splicing variants leads to DICER1 syndrome diagnosis
Source: Fam Cancer. 2023 May 30;22(4):487–93. doi: 10.1007/s10689-023-00336-1 (PMC10541835; doi:10.1007/s10689-023-00336-1)
Supplement: Supplementary file 1 — Supplementary file1 (DOCX 2632 kb) [file 10689_2023_336_MOESM1_ESM.docx]

**Supplementary Information**

**Reclassification of two germline *DICER1* splicing variants leads to DICER1 syndrome diagnosis**

Maria Apellaniz-Ruiz ^1*^, Nelly Sabbaghian ^2^, Anne-Laure Chong ^2^, Leanne de Kock ^3^, Semra Cetinkaya ^4^, Elvan Bayramoğlu ^4^, Winand N. M. Dinjens ^5^, W. Glenn McCluggage ^6^, Anja Wagner ^7^, Aslihan Arasli Yilmaz ^4^, William D. Foulkes ^2, 8, 9^

**Correspondence:** Dr. Maria Apellaniz-Ruiz, Genomics Medicine Unit, Navarrabiomed, Hospital Universitario de Navarra (HUN), Universidad Pública de Navarra (UPNA), IdiSNA. Calle Irunlarrea 3, 31008 Pamplona, Navarra, Spain. Phone number: +34 848428781.

Email: [mv.apellaniz.ruiz@navarra.es](mailto:mv.apellaniz.ruiz@navarra.es) / mariavalvanera.apellaniz@unavarra.es

**Index:**

- **Supplementary Material & Methods** ……………………………………….. **3**
  - **Participants and Samples** ….……..…………………………….………....... **3**
  - **Molecular screening** ……………………………………………………..….. **3**
  - ***In silico* splicing predictions** …………………………………………....…... **5**
  - ***In vitro* splicing assays - Mini-gene assays** .…………………………....…... **5**
  - ***In vitro* splicing assays - Patient derived mRNA analysis** ….………....….. **6**
- **Supplementary Data** ………………………………………………………….. **7**
  - ***DICER1* variant classification** ……………………….………………...……. **7**
- **Supplementary Figures** …………………………………………………….....**10**
  - **Supplementary Figure 1** …………………………………………………..... **10**
  - **Supplementary Figure 2** ……………………………………………………. **11**
  - **Supplementary Figure 3** ……………………………………………………. **12**
  - **Supplementary Figure 4** ……………………………………………………. **13**
  - **Supplementary Figure 5** ……………………………………………………. **14**
- **Bibliography** ……………………………………………………………..…... **15**

**Supplementary Material & Methods**

**Participants and samples**

The study followed the principles of the Declaration of Helsinki and it was approved by the McGill University Faculty of Medicine Institutional Review Board (A08-M61-09B). All participants and/or their legal guardians provided written informed consent to participate in the study.

Family/Case 1: Peripheral blood samples were collected from the proband, her mother and four of her siblings (two sisters and two brothers). Formalin-fixed, paraffin-embedded (FFPE) tissue was obtained from the SLCT and the FTND.

Family/Case 2: Peripheral blood samples were collected from proband and mother at the primary institution. Fibroblasts from the proband’s mother were obtained as well as DNA from the mother’s FTND and the proband’s thyroid nodule and ovarian tumor. Formalin-fixed, paraffin-embedded (FFPE) tissue was obtained from the brain sarcoma.

**Molecular screening**

Family 1: Germline DNA was extracted from blood samples using the Gentra Puregene Blood kit and from FFPE tumor tissue with QIAamp DNA FFPE Tissue Kit (Qiagen, ON, Canada). Tumour DNA was screened for *DICER1* mutations using a custom-design Fluidigm access array (Fluidigm, ON, Canada) [1]. Germline variants identified were PCR-amplified and Sanger sequenced to confirm the findings. In addition, germline DNA from the proband’s relatives was screened to evaluate variant segregation in the family.

Family 2: Germline DNA from the mother and daughter was extracted from blood samples using blood chemagic™ Kits (PerkinElmer). *PTEN* and *DICER1* testing was done by PCR amplification and Sanger sequencing. Mother-derived fibroblasts were obtained from a skin biopsy. The fibroblasts were cultured and utilized to perform RNA experiments (see below “Patient derived mRNA analysis” section). DNA from the mother’s FTND, the daughter’s thyroid nodules and ovarian tumor was extracted from tissue sections with high percentage (>80%) of tumor / lesional cells using lysis buffer with 5% Chelex 100 (BioRad) and proteinase K. In addition, DNA from the brain sarcoma was extracted from FFPE tissue with QIAamp DNA FFPE Tissue Kit (Qiagen, ON, Canada). Exons 24 and 25 of *DICER1* were PCR-amplified and Sanger sequenced to screen for somatic RNAse IIIb hotspot mutations. Before this study, as part of the clinical workup, the brain sarcoma was screened for somatic mutations using the gene panel Neuropanel V3.1. This panel is designed to analyze **1**) the coding sequence of *ATRX* (coverage by design 97%), *CDKN2A* (94%), *CDKN2B* (91%), *CI*C (81%), *DAXX* (93%), *DDX3X* (95%), *FUBP1* (97%), *NF1* (99%), *NF2* (100%), *PTCH1* (96%), *PTCH2* (92%), *PTEN* (94%), *SETD2* (96%), *SMO* (87%), *SUFU* (97%), *TRAF7* (72%) and *TP53* (98%); **2**) hotspot mutations in *ACVR1* (exon 6, 7, 8 and 9), *AKT* (3), *ALK* (20, 22, 23, 24, 25), *BRAF* (11, 15), *CTNNB1* (3), *EGFR* (3, 7, 15), *FGFR1* (12, 14, 15), *H3F3A* (2), *H3F3B* (2), *HIST1H3B* (1), *HIST1H3C* (1), *IDH1* (4), *IDH2* (4), *KLF4* (4) and *PIK3CA* (10, 21); as well as **3**) the *TERT* promoter and **4**) the copy number variation across the genome (SNPs located in chromosomes 1, 2, 3, 4, 6, 7, 8, 9, 10, 12, 16, 17, 19, 22 and X, detailed methodology see [2]). In addition, DNA methylation profiling was evaluated in tumor DNA with the Infinium MethylationEPIC BeadChip - 850k (detailed methodology described in [3, 4]) and translocation and gene fusions were investigated on tumor RNA with FusionPlex® Sarcoma v2 (Archer Dx, Boulder, CO). Sarcoma methylation classifier v10 was used to classify the brain sarcoma methylation group.

***In silico* splicing predictions**

We used *in silico* splicing prediction programs SpliceAI, Human Splicing Finder, NNSplice, NetGen2 and dbscSNV using AdaBoost or Random Forest algorithms to evaluate the impact of the germline *DICER1* variants. Links to the software can be found in the Bibliography section.

***In vitro* splicing assays**

1. **Mini-gene assays**

Mini-gene experiments were performed to model *DICER1* c.5365-4A>G germline variant following the protocol in Tompson *et al* [5]. Briefly, genomic DNA from case 1 was PCR amplified using primers 5’-ctgactgaCTCGAGAATGGGGTGGGGATATTTTT-3’ and 5’-tcagtcagGGATCCAAATCTGACAACAGCACACCA-3’. The region amplified included part of the sequence of intron 24, exon 25, intron 25, exon 26 and part of intron 26 (fragment of 683 bases). After confirmation of a single fragment, the PCR products were digested with XhoI & BamHI, purified with PureLink PCR Purification Kit (ThermoFisher Scientific, ON, Canada) and cloned into pSPL3 exon trapping vector (Invitrogen, Carlsbad, CA) using T4 ligase (NEB, MA, USA). The DICER1-pSPL3 vectors were transformed into chemically competent E. Coli cells (One Shot™ TOP10 from ThermoFisher Scientific) and grown on LB plates using ampicillin. Several bacterial clones were picked and propagated, and plasmid DNA was purified using E.Z.N.A. Plasmid DNA Mini and Midi Kits (Omega BioTech, Georgia, USA). Plasmid DNA was then analysed by Sanger sequencing to identify wild type (wt) and mutant (mut) constructs and to rule out any sequence deviations generated by PCR.

Human Embryonic Kidney cells, HEK293T were transfected with “*DICER1* wt” and “*DICER1* c.5365-4A>G” pSPL3 minigenes. HEK293T cells were cultured in DMEM medium (Gibco Laboratories, Gaithersburg, MD, USA) supplemented with 10% fetal bovine serum (Gibco) and 1X PenStrep (Gibco). The cells were transfected using 250 μl OptiMEM (Gibco), 15 μl Lipofectamine 2000 (ThermoFisher Scientific) and 2,5 μg pSPL3 vector DNA. Total RNA was extracted 24h- after transfection using Trizol method (ThermoFisher Scientific) and reverse transcribed into cDNA with SuperScript IV First-Strand Synthesis System and random hexamers (Thermo Fischer Scientific). Thereafter, PCR was performed on cDNA using pSPL3 specific primers 5’-TCTGAGTCACCTGGACAACC-3’ and 5’-ATCTCAGTGGTATTTGTGAGC-3’. The PCR products were separated on agarose gels and Sanger sequenced.

1. **Patient derived mRNA analysis**

Patient-derived lymphocytes (case 1) and fibroblasts (mother of case 2) were grown in Iscove’s Modified Dulbecco’s Media (Wisent Bio Products) 5 x 10^6^ cells were treated with 28 ug/ml cycloheximide (Sigma Aldrich) or Dimethyl Sulfoxide (Sigma Aldrich) as control, in triplicate for 3 hrs at 37^o^ C, to evaluate the inhibition of nonsense mediated decay. Total RNA was extracted from lymphocytes and fibroblasts using Trizol Reagent (Thermo Fisher Scientific). RNA was reverse transcribed into cDNA with SuperScript III First-Strand Synthesis System and random hexamers (Thermo Fischer Scientific). PCR was performed on cDNA to amplify the region extending from exon 24 to 27. PCR reactions were separated on 1.8% agarose gels and each band was obtained using QIAquick Gel Extraction Kit (Qiagen), followed by Sanger sequencing.

**Supplementary Data**

***DICER1* variant classification**

Variant interpretation can be challenging and discrepancies may occur. To help with this task, the American College of Medical Genetics and Genomics and the Association for Molecular Pathology (ACMG-AMP) have developed standards and guidelines for the classification of sequence variants using criteria informed by expert opinion and empirical data [6]. In addition, gene-/disease-specific classification systems are being developed by expert panels, given that the applicability and weight assigned to certain criteria may vary by gene and disease. Such is the case for DICER1 [7]. Specific modifications to ACMG-AMP criteria include somatic tumour testing to identify RNase IIIb hotspot second hits and a system ranking DICER1 phenotype specificity (e.g. individuals with high-specificity phenotypes are much more likely than those with low-specificity phenotypes to have a germline pathogenic/likely pathogenic *DICER1* variant).

We have followed the ClinGen DICER1 and miRNA-Processing Gene Expert Panel Specifications to the ACMG/AMP Variant Interpretation Guidelines for DICER1 to classify the pathogenicity of the germline *DICER1* intronic variants [6, 7].

The criteria met by *DICER1* c.5365-4A>G is:

- PS3: Well-established *in vitro* functional studies supportive of a damaging effect on the gene or gene product (i.e. intronic variant found to have an out-of-frame impact on splicing via RNA assay)
- PS4_Supporting: The prevalence of the variant in affected individuals is significantly increased compared to the prevalence in controls (i.e. Case 1 with two moderate-specificity phenotypes, FTND and SLCT).
- PM2_Supporting: Absent from controls. Allele frequency <0.000005 across gnomAD (non-cancer) with no more than one allele in any subpopulation and at least 20x coverage (i.e. variant not present in gnomAD v3.1.2, region mean depth of coverage 30x, accessed in January 2023).
- PP3: Multiple lines of computational evidence support a deleterious effect on the gene or gene product:
  - SpliceAI: Splice-Altering, Acceptor Gain (Score = 0.98)
  - dbscSNV Ada: Deleterious (Score = 1)
  - dbscSNV RF: Deleterious (Score = 0.98)
  - Human Splicing Finder: MaxEnt Acceptor Site – broken wild type acceptor site (Variation= 7.2 > 3.84 🡪 -46.67%) and gained new acceptor splice site (Variation= -3.67 > 5.08 🡪 238.42%)
  - NNSplice: Acceptor site changed
  - NetGen2: Acceptor Site Gain
- PP4: Patient’s phenotype or family history is highly specific for a disease with a single genetic etiology (i.e. somatic testing revealed c.5125G>A (p.D1709N) in the FTND and c.5113G>A (p.E1705K) in the SLCT, as well as retention of the germline variant).

This variant meets one pathogenic strong and four pathogenic supporting criteria. This results in 8 points, classifying the variant as *Likely pathogenic*. Prior to performing RNA assays (PS3 criteria), the classification would be *variant of uncertain significance* (4 points).

The criteria met by *DICER1* c.5527+3A>G is:

- PS3: Well-established *in vitro* functional studies supportive of a damaging effect on the gene or gene product (i.e. intronic variant found to have an out-of-frame impact on splicing via RNA assay).
- PS4_Moderate: The prevalence of the variant in affected individuals is significantly increased compared to the prevalence in controls (i.e. Case 2 with two moderate-specificity phenotypes, thyroid nodules and retiform SLCT, and one low-specificity, brain sarcoma; and one individual diagnosed with a high-specificity phenotype, cystic nephroma, described in the literature [8]).
- PM2_Supporting: Absent from controls, Allele frequency <0.000005 across gnomAD (non-cancer) with no more than one allele in any subpopulation and at least 20x coverage (i.e. variant not present in gnomAD v3.1.2, region mean depth of coverage 30x, accessed in January 2023).
- PP3: Multiple lines of computational evidence support a deleterious effect on the gene or gene product:
  - SpliceAI: Splice-Altering, Donor Loss (Score = 0.65)
  - dbscSNV Ada: Deleterious (Score = 1)
  - dbscSNV RF: Deleterious (Score = 0.8)
  - Human Splicing Finder: MaxEnt Donor Site – broken wild type donor site (Variation= 5.64 > -1.07 🡪 -118.9%)
  - NNSplice: No changes detected
  - NetGen2: No changes detected
- PP4: Patient’s phenotype or family history is highly specific for a disease with a single genetic etiology (i.e. somatic testing revealed c.5439G>T (p.E1813D) hotspot mutation was detected in the retiform SLCT as well as retention of the germline variant).

This variant meets one pathogenic strong, one pathogenic moderate and three pathogenic supporting criteria. This results in 9 points, classifying the variant as *Likely pathogenic*. Prior to performing RNA assays (PS3 criteria), the classification would be *variant of uncertain significance* (5 points).

**Supplementary Figures**

**Supplementary Figure 1. Minigene assay results for *DICER1* c.5365-4A>G.**

**
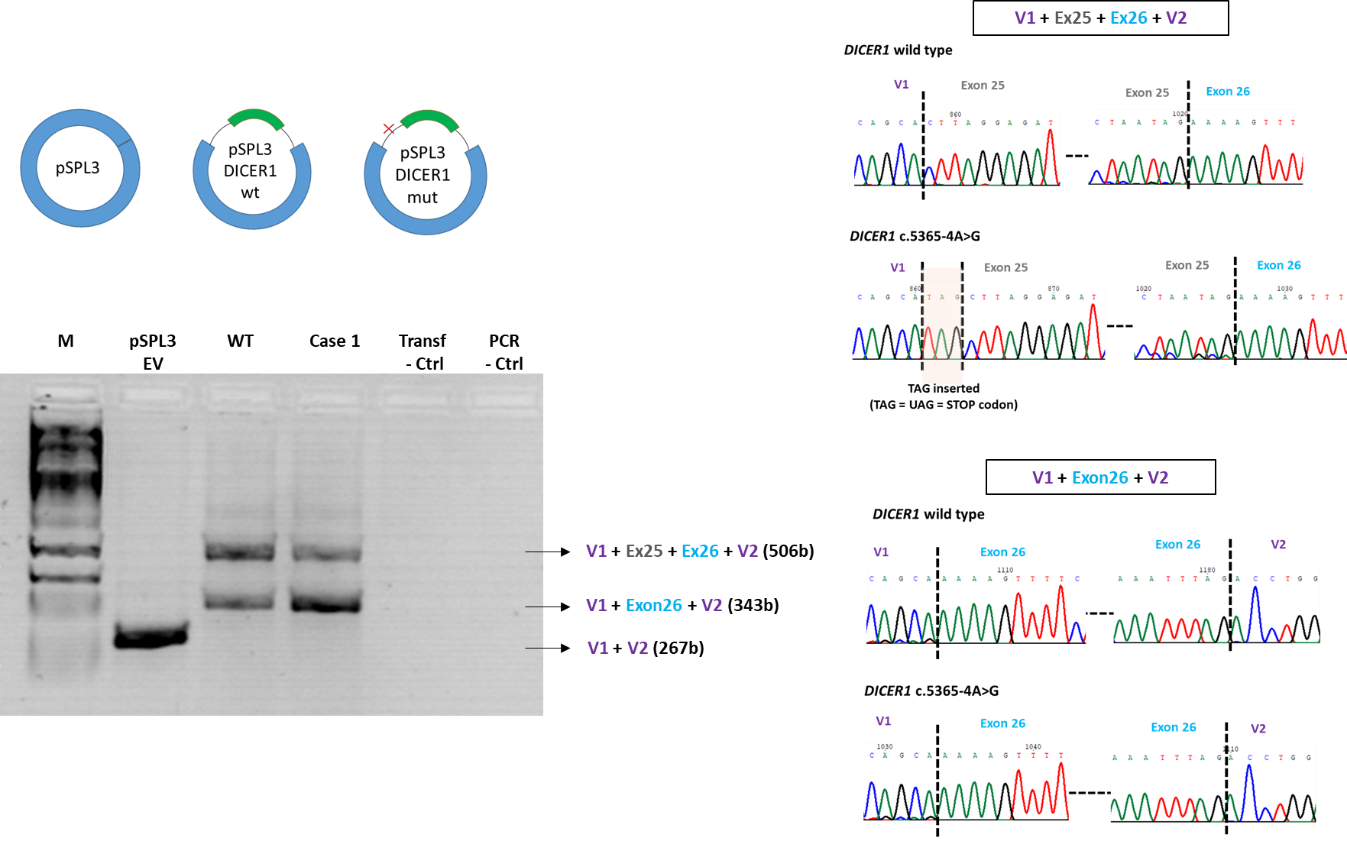
**

Agarose gel showing the RT–PCR products of introduced pSPL3 constructs and sequencing results. The experiments demonstrated that c.5365-4A>G resulted in two aberrant transcripts, one similar to the canonical transcript with the insertion of three bases (TAG) and a second altered transcript lacking exon 25. pSPL3 EV, pSPL3 empty vector; WT, pSPL3 containing *DICER1* wild-type sequence (intron24-exon25-intron25-exon26-intron26); Case 1, pSPL3 containing c.5365-4A>G, V1 & V2, exons from pSPL3 vector. Original image of the agarose gel is presented in Supplementary Figure 5.

**DICER1* wild type construct also produced a small amount of transcript lacking exon 25. This has already been observed using the same pSPL3 vector and modelling a different variant in *DICER1* exon 25 [9]. It may be an artefact of the assay. This is not observed when analyzing RNA extracted from blood from a healthy individual.

**Supplementary Figure 2. Case 1 mRNA derived analysis.**


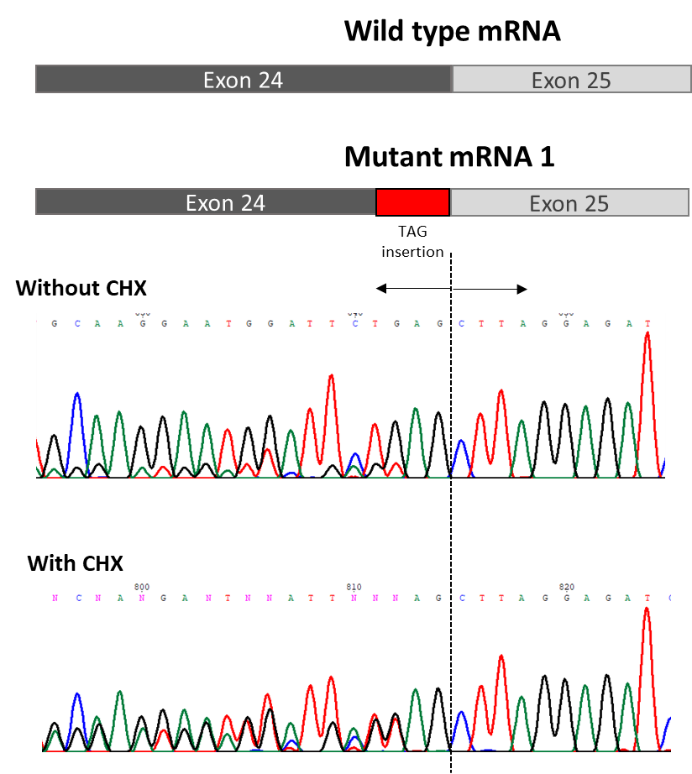


Chromatograms showing the sequences of the RT-PCR products from the upper band in the agarose gel. The band contains a mix of wild type and aberrant mRNAs. The addition of cycloheximide to the cultured lymphocytes inhibits nonsense mediated decay mechanism and seems to result in an increase of the aberrant mRNA, although Sanger sequencing is not quantitative.

**Supplementary Figure 3. Images of the agarose gel showing RT–PCR products in Case 1.**

**
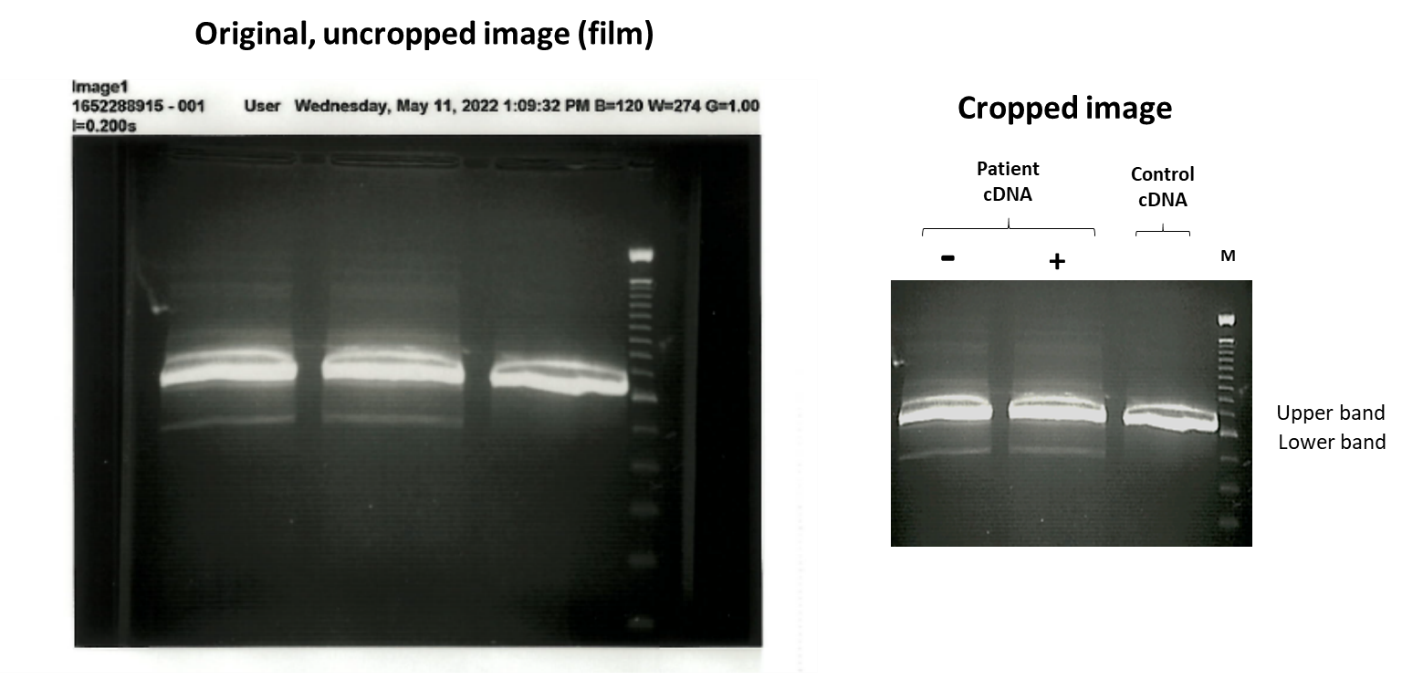
**

Original uncropped image of the agarose gel showing the RT–PCR products detected in Case 1 (left side) and cropped image of the original used in Figure 2A (right side).

**Supplementary Figure 4. Images of the agarose gel showing RT–PCR products in Case 2.**

**
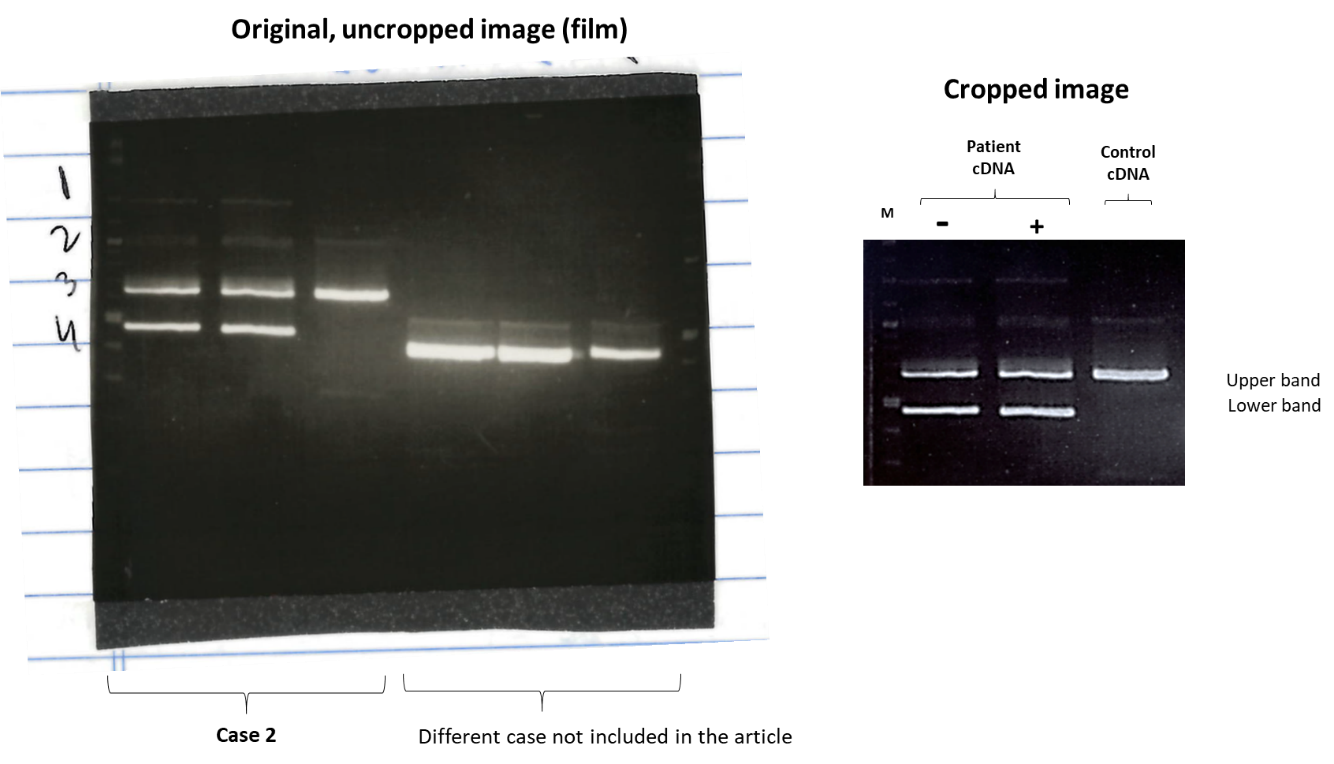
**

Original uncropped image of the agarose gel showing the RT–PCR products detected in Case 2 (wells 1 to 3) and in a different case not included in this study (wells 4 to 6) [left side], and cropped image of the original used in Figure 2B [right side].

**
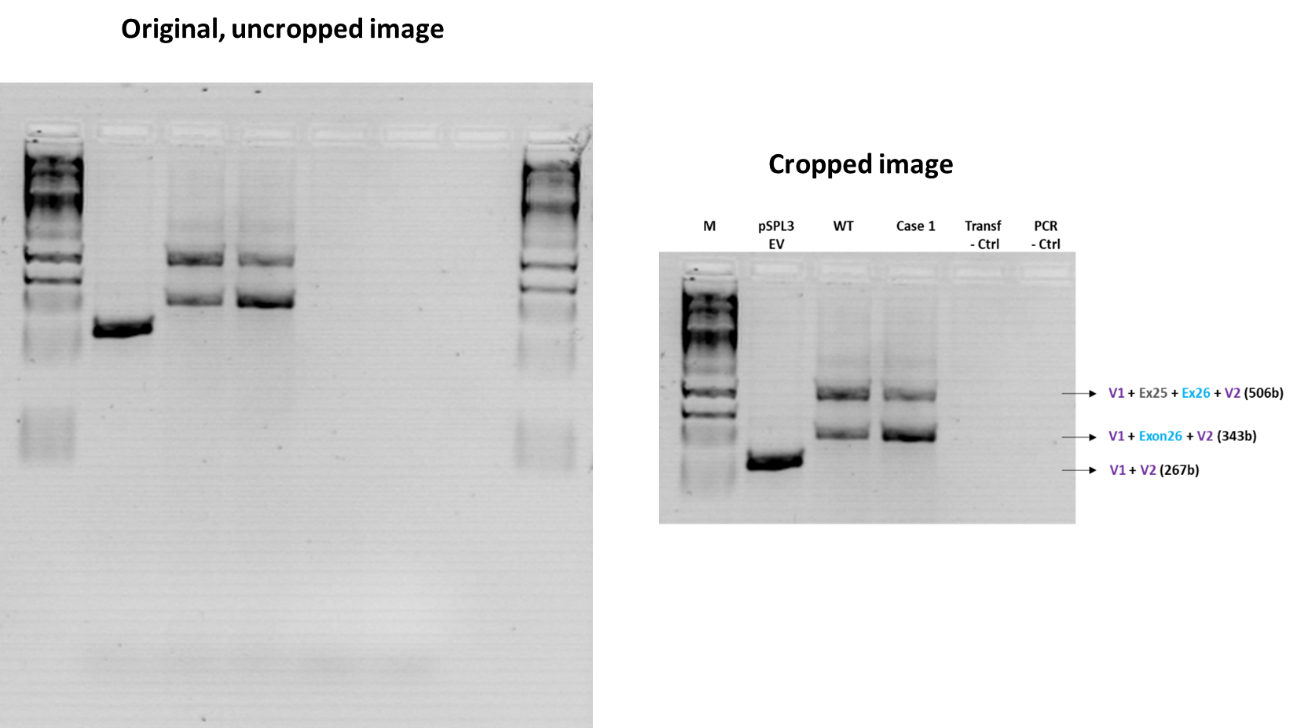
Supplementary Figure 5. Images of the agarose gel of the minigene assay for *DICER1* c.5365-4A>G.**

Original uncropped image of the agarose gel showing the RT–PCR products of introduced pSPL3 constructs (left side), and cropped image of the original used in Supplementary Figure 1 (right side).

**Bibliography:**

1. de Kock L et al (2014) Pituitary blastoma: a pathognomonic feature of germ-line DICER1 mutations. Acta Neuropathol. 128:111-122. https://doi.org/10.1007/s00401-014-1285-z

2. Dubbink HJ et al (2016) Diagnostic Detection of Allelic Losses and Imbalances by Next-Generation Sequencing: 1p/19q Co-Deletion Analysis of Gliomas. The Journal of molecular diagnostics : JMD 18:775-786. https://doi.org/10.1016/j.jmoldx.2016.06.002

3. Koelsche C et al (2021) Sarcoma classification by DNA methylation profiling. Nat Commun 12:498. https://doi.org/10.1038/s41467-020-20603-4

4. Koelsche C et al (2019) Genome-wide methylation profiling and copy number analysis in atypical fibroxanthomas and pleomorphic dermal sarcomas indicate a similar molecular phenotype. Clin Sarcoma Res 9:2. https://doi.org/10.1186/s13569-019-0113-6

5. Tompson SW & Young TL (2017) Assaying the Effects of Splice Site Variants by Exon Trapping in a Mammalian Cell Line. Bio Protoc 7. https://doi.org/10.21769/BioProtoc.2281

6. Richards S et al (2015) Standards and guidelines for the interpretation of sequence variants: a joint consensus recommendation of the American College of Medical Genetics and Genomics and the Association for Molecular Pathology. Genet. Med. 17:405-424. https://doi.org/10.1038/gim.2015.30

7. Hatton et al (2023) Specifications of the ACMG/AMP Variant Classification Guidelines for Germline DICER1 Variant Curation. Hum Mutat. https://doi.org/10.1155/2023/9537832

8. Doros LA et al (2014) DICER1 mutations in childhood cystic nephroma and its relationship to DICER1-renal sarcoma. Mod. Pathol. 27:1267-1280. https://doi.org/10.1038/modpathol.2013.242

9. Wu MK et al (2013) Biallelic DICER1 mutations occur in Wilms tumours. J. Pathol. 230:154-164. https://doi.org/10.1002/path.4196

**Additional resources:**

SpliceAI: <https://spliceailookup.broadinstitute.org/>

Human Splicing Finder: <https://www.genomnis.com/access-hsf>

NNSplice: <https://www.fruitfly.org/seq_tools/splice.html>

NetGen2: <https://services.healthtech.dtu.dk/service.php?NetGene2-2.42>

dbscSNV: <http://www.liulab.science/dbscsnv.html>
